# Pupillary Light Reaction during High Altitude Exposure

**DOI:** 10.1371/journal.pone.0087889

**Published:** 2014-02-04

**Authors:** Maximilian Schultheiss, Kai Schommer, Andreas Schatz, Barbara Wilhelm, Tobias Peters, M. Dominik Fischer, Eberhart Zrenner, Karl U. Bartz-Schmidt, Florian Gekeler, Gabriel Willmann

**Affiliations:** 1 Center of Ophthalmology, University of Tübingen, Tübingen, Germany; 2 Nuffield Laboratory of Ophthalmology Oxford, University of Oxford, Oxford, United Kingdom; 3 Department of Sports Medicine of Medical Clinic, University of Heidelberg, Heidelberg, Germany; 4 Department of Ophthalmology, Katharinenhospital, Stuttgart, Germany; Justus-Liebig-University Giessen, Germany

## Abstract

**Purpose:**

This study aimed to quantify the pupillary light reaction during high altitude exposure using the state of the art Compact Integrated Pupillograph (CIP) and to investigate a potential correlation of altered pupil reaction with severity of acute mountain sickness (AMS). This work is related to the Tübingen High Altitude Ophthalmology (THAO) study.

**Methods:**

Parameters of pupil dynamics (initial diameter, amplitude, relative amplitude, latency, constriction velocity) were quantified in 14 healthy volunteers at baseline (341 m) and high altitude (4559 m) over several days using the CIP. Scores of AMS, peripheral oxygen saturation and heart rate were assessed for respective correlations with pupil dynamics. For statistical analysis JMP was used and data are shown in terms of intra-individual normalized values (value during exposure/value at baseline) and the 95% confidence interval for each time point.

**Results:**

During high altitude exposure the initial diameter size was significantly reduced (p<0.05). In contrast, the amplitude, the relative amplitude and the contraction velocity of the light reaction were significantly increased (p<0.05) on all days measured at high altitude. The latency did not show any significant differences at high altitude compared to baseline recordings. Changes in pupil parameters did not correlate with scores of AMS.

**Conclusions:**

Key parameters of the pupillary light reaction are significantly altered at high altitude. We hypothesize that high altitude hypoxia itself as well as known side effects of high altitude exposure such as fatigue or exhaustion after ascent may account for an altered pupillogram. Interestingly, none of these changes are related to AMS.

## Introduction

High altitude exposure and thus hypoxia effects the central nervous system [1] leading to an impairment of arithmetic, language, perception, memory, learning and psychomotor skills [2], [3], [4], [5]. Furthermore, many people who ascend to high altitude suffer from high-altitude headache (HAH), which presents the most common neurological symptom during high altitude exposure and the most characteristic clinical feature of acute mountain sickness (AMS) [1]. Other vegetative symptoms of AMS may include nausea, insomnia, anorexia and dizziness [6]. Acute mountain sickness can only be diagnosed by using a standardized questionnaire and objective parameters for assessment of AMS are still absent [7]. Despite the well-known clinical presentation of AMS, it is still not possible to predict who will suffer from AMS at high altitude. Only risk factors such as rapid ascent, exertion, history of altitude illness, young age and genetic predisposition are known [1]. Furthermore, the underlying pathomechanism for the development of AMS is still unclear and currently debated [1].

The pupil presents a gateway to the brain. Its diameter is mainly influenced by the amount of light entering the eye and is controlled by the pupillary light reflex [8]. Additionally, a central inhibition of the parasympathetic input to the pupil reaction by the sympathicus exists [9], [10], [11]. The autonomous nervous system is part of the central nervous system and receives further input from large parts of the brain; this is the reason, why pain or cognitive tasks can also influence the pupil [12], [13], [14]. Furthermore the pupil is influenced by the level of alertness or sleepiness [15], the near response via the occipital cortex [16], psychosensory reactions [12], [13], [17] and by age [8] presenting a complex interaction of different parts of the brain.

Two recent studies have investigated pupillary light reactions during high altitude exposure using different devices and exposure protocols to high altitude [18], [19]. Both studies showed significant altitude related changes in pupillary light reaction as a sign of altered autonomous regulation. Regarding the correlation of pupil dynamics with severity of AMS, which presents with many clinical symptoms of the central nervous system, both studies showed inconsistent results [18], [19].

Because of these inconsistencies in the studies so far we assessed pupil dynamics during high altitude exposure using the state of the art Compact Integrated Pupillograph (CIP by AMTech, Germany). Furthermore, as the exposure protocols of the two earlier studies were not designed to investigate whether these pupillary changes are related to AMS or not, we used a well-established exposure protocol especially designed to assess AMS related correlations [20].

## Materials and Methods

### Study design

**Figure 1:**
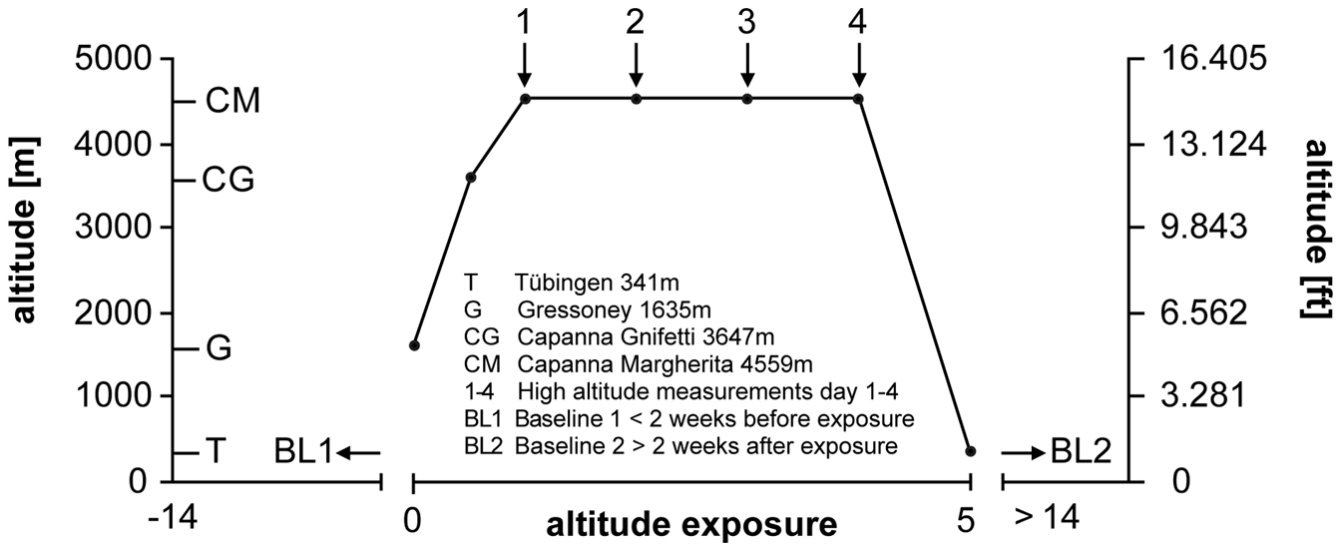
Ascent profile. The graph represents the ascent profile of volunteers to the research facility of the Capanna Margherita (4559 m). Numbers indicate all four measurements using the CIP at high altitude. Before and after the ascent to the Capanna Margherita baseline measurements were performed.

In this prospective field study 14 healthy volunteers (7 females and 7 males; age 25–54, mean 35±4 years) ascended to the Capanna Margherita 4559 m (CM; Valais Alps, Italy). The participants were accompanied by a mountain guide according to the ascent profile in figure 1: day0 from Gressoney (Italy) 1635 m to Punta Indren 3260 m by cable car followed by 2 hours (hrs) of hiking to the Capanna Gnifetti 3647 m; day1 ascent to the CM in 4–6 hrs. All participants started in Gressoney and arrived at the CM within 24 hrs; glacier-goggles were worn to protect the eyes from bright UV-light exposure at all times during ascent and outside the CM. The volunteers (14) spent 3 nights (from day1 to day4) at the CM. Prior to baseline recordings in Tübingen (341 m) (BL1 =  before and BL2 =  after the expedition) all participants had to spend at least 14 days below 2000 m. As general exclusion criteria none of the participants had any type of cardiac or respiratory diseases, a history of high altitude cerebral edema or high altitude pulmonary edema. Ophthalmological exclusion criteria were refractive error greater than ±5 diopters spherical equivalent, astigmatism more than ±2 diopters, presence of disorders affecting the iris, or optical opacities limiting imaging quality. During the entire study period only non-steroidal anti-inflammatory agents were allowed when administered by our group physicians.

According to the tenets of the Declaration of Helsinki 1975 (1983 revision) the investigation was planned and performed. The study protocol had been approved by the Ethics Committee of the Medical Faculty at the University of Tübingen (project number: 258/2010B01) prior to the start of the study. Furthermore all participants were informed about the aims of the study, the study protocol and gave written informed consent prior to the start of the research expedition.

### Clinical assessment of acute mountain sickness (AMS) and clinical parameters

To evaluate AMS the Lake-Louise (LL) and the AMScerebral score (AMS-C) of the Environmental Symptom Questionnaire (ESQ III) were used [21], [22]. Scores were assessed once at BL1 and BL2, and at high altitude twice a day. Participants were thoroughly informed regarding the nature of the questionnaires at the beginning of the study (prior to baseline recordings). Cut-off criterion for the LL was a score ≥5 in the presence of headache and for AMS-C ≥0.70. These cut-off criteria have previously been used and AMS was assumed when both scores fulfilled the cut-off criteria [23]. Oxygen saturation (spO2) and heart rate (HR) were measured once at BL1 and BL2 and at high altitude before getting up in the morning with a finger pulse oxymeter (oxy control 4c®, Geratherm Medical AG, Geschwenda, Germany). All measurements were performed by the same staff in the identical order daily.

### Pupillography

All measurements were performed under dark conditions in the morning, before the subjects went outside the CM. Only on day1 - due to the ascent to the CM – measurements were performed in the evening after >6 hrs of rest. Before the recording the noise was reduced to a minimum and subjects were adapted to darkness for 5 minutes.

For pupillography the state of the art CIP (AMTech, Dossenheim, Germany) was used. The stationary CIP measured the pupil diameter with a CCD infrared line camera. The head of the examined subject was positioned, the pupil focused with a camera and a standardized stimulus of 200 msec duration with a corneal intensity of 3 lx was applied; consecutive recordings were then performed automatically by the CIP. In each session, measurements were repeated five times (Fig. S1), artifact measurements were deleted and afterwards an average value was automatically calculated. The average pupillogram was used to calculate the initial diameter, the amplitude, the relative amplitude, the latency and the constriction velocity. The constriction velocity was calculated by measuring the gradient between 40% and 80% of the constriction amplitude. A detailed description of all parameters is provided in the table S1.

### Statistical methods

For statistical analysis JMP (Version 8.0.2, SAS Institute, Cary, NC) was used. Comparisons were performed by multivariate analysis of variance (MANOVA) for repeated measures. Data are shown in terms of intra-individual normalized values to baseline 1 (value during exposure/value at BL1) and the 95% confidence interval for each time point. To evaluate a possible correlation between changes of pupil parameters and AMS or basic physiologic parameters the Pearson's correlation coefficient between recorded values and clinical parameters (SpO2, HR, AMS-C, LL) was calculated.

## Results

### Clinical examination and AMS scores

In the whole cohort the mean of AMS-C and LL scores were 0 at BL1 and BL2. On day2 AMS-C was 1.06±0.77 (mean ± standard deviation (SD)) and LL respectively 5.2±2.7. Heart rate increased at high altitude from 60±7 min^−1^ at BL1 to 83±11 min^−1^ on day2 and SpO2 decreased from 98.5±1.3% at BL1 to 71.5±5.6% on day2 in the whole cohort. According to our cut-off criteria for AMS the AMS+ and the AMS− group contained of seven volunteers each. Compared to the AMS− group the AMS+ group showed a greater mean in AMS-C and LL on day2, a increased HR and a decreased SpO2 (means ± SD for the AMS+ group: AMS-C 1.62±0.59; LL 6.7±2.1; HR 89±8 min-1; SpO2 69.9±6.2%; means ± SD for the AMS− group: AMS-C 0.40±0.19; LL 3.5±2.2; HR 77±9 min-1; SpO2 73.5±4.6%).

### Initial diameter

**Figure 2:**
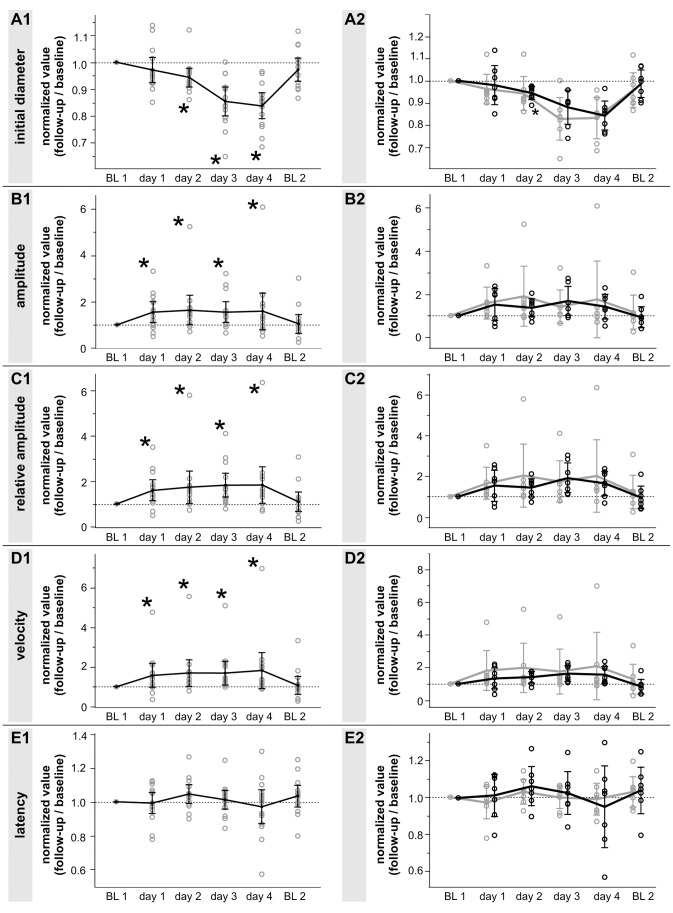
Pupil changes at high altitude. Graphs represent the intraindividual changes in pupil parameters (expressed as normalized value to the baseline measurement before exposure, BL1) of the whole cohort (A1–E1; all graphs on the left side) and of the AMS+ respectively AMS− group (A2–E2; all graphs on the right side). The AMS+ group is represented by the black curve and the AMS− group by the gray curve. Displayed are the changes in initial diameter (A1, A2), amplitude (B1, B2), relative amplitude (C1, C2), latency (D1, D2) and contraction velocity (E1, E2). Data are depicted as mean normalized value ± the 95% confidence interval for each time point with * p<0.05; number of subjects: n = 14 (A1–E1) and n = 7 for AMS+ and AMS− group (A2–E2).

**Figure 3:**
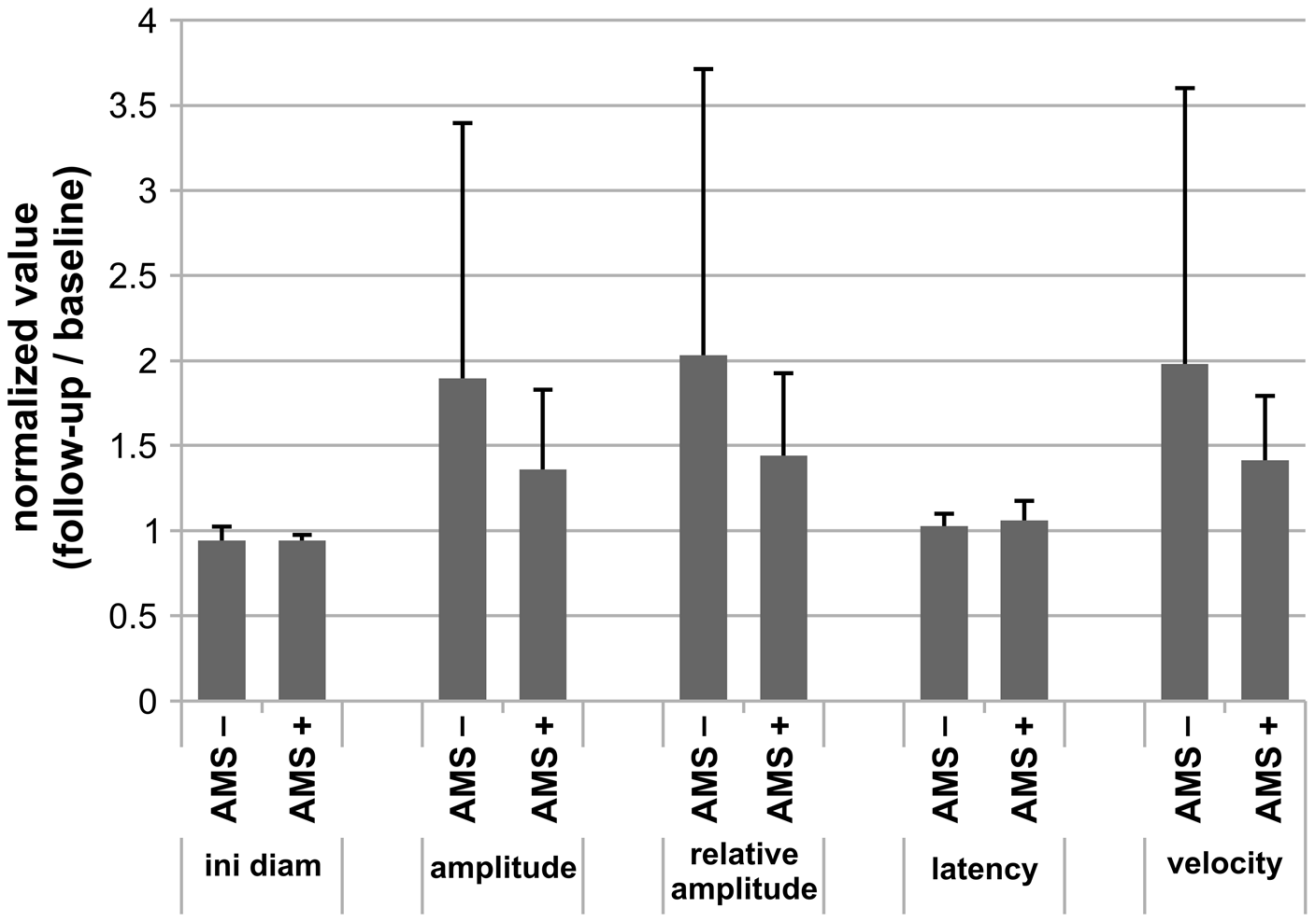
Pupil changes in the subgroups of AMS+ and AMS− at high altitude. Bar graphs represent intraindividual changes in pupil parameters (expressed as normalized values to the baseline measurements before exposure, BL1) of the AMS+ respectively AMS− group on day2. Displayed are the changes in initial diameter, amplitude, relative amplitude, latency and contraction velocity. Data are depicted as mean normalized value ± SD; number of subjects: n = 7 for AMS+ and AMS− group.

The normalized value of the overall initial diameter was significantly reduced for all time points at high altitude (Fig. 2A1). The normalized value of the initial diameter decreased slowly over time at high altitude and increased back to comparable values of BL1 on BL2 (means of the initial diameter ± SD: BL1: 6.92±0.94, day1: 6.71±0.93, day2: 6.52±0.91, day3: 5.89±0.94, day4: 5.79±0.94, BL2: 6.72±1.04). After the division of the whole cohort into subjects with and without AMS, both subgroups showed a decrease in initial diameter similar (Fig. 2A2). The mean of both groups on day2 were close to each other, but the AMS+ group showed a smaller SD (means of the initial diameter ± SD: AMS+0.95±0.03; AMS− 0.94±0.08) (Fig. 3).

### Amplitude and relative amplitude

Normalized values of amplitude and relative amplitude showed a significant increase during high altitude exposure on all days tested (p<0.05)(Fig. 2B1 and 2C1). In detail, the mean of the amplitude and relative amplitude increased already on the first day by 1.30 and 1.38 folds, respectively. Throughout the time spent at high altitude absolute and relative amplitude of the light reaction stayed at this elevated level (mean of the amplitude ± SD: BL1 0.71±0.4, day1 0.96±0.49, day2 0.92±0.40, day3 0.89±0.36, day4 0.82±0.32, BL2 0.68±0.41; mean of the relative amplitude ± SD: BL1 10.62±6.47, day1 14.66±7.59, day2 14.56±7.04, day3 15.52±6.69, day4 14.71±6.21, BL2 10.69±6.77) (Fig. 2B1 and 2C1). After the division of the cohort into groups with and without AMS normalized values for both parameters showed the same trend, but the AMS+ group changed slightly slower than the AMS− group (Fig. 2C2).

On day2 the mean of the amplitude and relative amplitude were greater in the AMS− compared to the AMS+ group (mean of the amplitude ± SD: AMS+1.36±0.47; AMS− 1.9±1.5; mean of the relative amplitude ± SD: AMS+1.44±0.48; AMS− 2.03±1.68) (Fig. 3).

### Latency and constriction velocity

Both temporal parameters showed different results. The latency did not show any significant differences on all days tested at high altitude compared to BL1 in the overall group-analysis (Fig. 2D1 (means of the latency of the whole group ± SD: BL1 0.29±0.03, day1 0.29±0.03, day2 0.30±0.02, day3 0.39±0.02, day4 0.28±0.04, BL2 0.30±0.02).

In contrast, the constriction velocity showed a significant increase at all time points tested during high altitude exposure compared to BL1 (means of the constriction velocity ± SD: BL1 2.74±1.29, day1 3.64±1.50, day2 3.73±1.15, day3 3.76±1.13, day4 3.99±2.0, BL2 2.62±1.46) (Fig. 2E1). After the division of the cohort into volunteers with and without AMS values in both groups increased, but the constriction velocity of the AMS+ group showed a slower increase compared to the AMS− group (Fig. 2E2). Consequently the mean of constriction velocity in AMS− was higher compared to AMS+ on day2 (mean of the constriction velocity ± SD: AMS+ 1.41±0.38; AMS− 1.98±1.62) (Fig. 3).

### Correlations to AMS and clinical parameters

No significant correlations of AMS-C, LL, spO2 or HR were observed with any pupil parameter (data not shown).

## Discussion

Our study demonstrates that high altitude exposure results in significant changes of the pupillary light reaction. We found, that the initial pupil diameter significantly decreased and the amplitude, relative amplitude and velocity significantly increased during high altitude exposure (Fig. 2). However, none of the pupillometric parameters correlated with LL or AMS-C. In the subgroup analysis the AMS− group showed slightly greater changes during the first two days at high altitude compared to the AMS+. Nevertheless both subgroups showed the same trend independent of AMS (Fig. 2).

In comparison with two other studies investigating the effects of high altitude on pupil dynamics it has to be emphasized that considerable differences between the devices used, the exposure protocol, the method of analysis of the pupillograms, the settings during measurement and the state of exertion of the participants existed. All these factors may have had an impact on the pupillogram and possibly account for the different outcome between the studies.

The FIT 2000 screener used by Cymerman et al. and our CIP are stationary and fully automated pupillometers with an accuracy of 0.05 mm [19]. The pupillometer used by Wilson et al. is a hand-held device and measures the pupil diameter with an accuracy of 0.1 mm [18], [24]. It is of interest that a hand-held pupillometer can easily be tilted and therefore may influence the measurement. Nevertheless all pupillometers showed good reliability and reproducible results [18], [19].

The methodology of pupillography in both recent studies was not described in full detail making it difficult to compare the studies with each other. In the paper of Wilson et al. it is not stated if several or only a single measurement was performed for each time point [18]. Cymerman et al. performed four measurements at each time point and the mean of three measurements was used for analysis [19]. This is an important aspect as variability of the light response is high even under stable conditions and that is why it is recommended to perform several measurements at a certain time point and calculate an average [8].

Artifact management is of key importance when pupillograms are recorded as artifacts may strongly influence the calculated average pupillogram. However, in both recent studies it was not stated if a respective artifact management was used.

In addition the ambient illumination may also influence the study outcome. In the study of Cymerman et al. the ambient illumination was specified for recordings at high altitude, but not for baseline recordings [19]. Furthermore an illumination of 230–245 lx may present a rather bright surrounding for pupillography [19]. In the study of Wilson et al. the ambient illumination was measured by the pupillometer and was kept constant with measurements performed in a shaded room and darkened tent [18]. In contrast to Wilson et al. we used a completely darkened room and let the volunteers adapt to the darkness for 5 minutes before recordings were performed to minimize potential confounding effects despite the rationale that dark adaptation is not mandatory for pupillography at all.

The latency, amplitude and constriction velocity largely depend on the intensity of the light stimulus applied. Unfortunately in both recent studies the duration but not the intensity of the flash was described [18], [19].

The ascent profile and therefore the degree of exertion of volunteers varied to a great extent between all three studies. In the study of Wilson et al. subjects were brought by airplane from 300 m up to 3450 m before trekking to a maximum altitude of 4770 m for 8 days [18]. In the study of Cymerman et al. subjects were brought by car from sea-level to 4300 m altitude breathing 100% bottled oxygen for the whole time of transport and no trekking or other form of exercise was performed at high altitude [19]. In our study subjects started from 1635 m, ascended by cable car to 3260 m and then hiked to the CM at 4559 m within 24 hrs where they stayed for the following four days until descent (Fig. 1).

Although many differences in the methodology existed between all three studies and results regarding amplitude, latency and constriction velocity differed, a reduction of the initial diameter was consistently noted. As humans at high altitude suffer from sleep disturbances and daytime sleepiness a decreased pupil diameter may indicate for a decreased central nervous activation level and daytime sleepiness respectively [1], [25]. The latter causes a disinhibition of the parasympathetic Edinger Westphal nuclei resulting in a smaller pupil diameter. As another hint for an increased parasympathetic activation volunteers suffering from AMS showed vegetative symptoms like nausea, anorexia and dizziness [6]. Although the parasympathicus seems to be activated there is also an increase in catecholamines and stress hormones like cortisol during high altitude exposure [19], [26], [27]. Unfortunately there is no profound understanding about the parasympathetic tone at high altitude. However, to gain deep insight about the sympathetic and parasympathetic interaction the analysis of spontaneous pupillary movements in darkness would be of special interest as they reflect the level of tonic central nervous activation [15] and may confirm the hypothesis of increased daytime sleepiness. As AMS is related to many vegetative symptoms a correlation between AMS and spontaneous pupillary movements may exist and has been suggested previously [19].

It is of further interest that a swelling of the optic disc occurs during high altitude exposure as reported previously [28]. However, as differences in pupil dynamics result in a slight miosis it is unlikely that optic disc edema is the cause as this would result in a mydriasis.

It is also unlikely that hypoxia itself is solely responsible for the miosis as SpO2 initially dropped after arrival at the CM and then increased over time spent at high altitude in contrast to the pupil diameter, which decreased continuously. Nevertheless Cymerman et al. showed that 3 hrs of hypoxia is sufficient to decrease the pupil diameter significantly [19].

Furthermore, it is important to note that other factors such as cold temperature, fatigue or exhaustion after ascent may also influence the outcome of the pupillography recording in addition to the effect of hypoxia at high altitude.

In our study none of the investigated pupillographic parameters were related to AMS and this finding is in line with Wilson et al. [18] while in contrast to Cymerman et al. who showed a significant correlation of the latency to AMS [19].

We conclude that a decrease in pupil diameter was the only parameter consistently altered in all three studies and propose that the most likely reason for the miosis may be an increase in parasympathetic tone and thus the result of high altitude exposure. In addition to a decreased pupil diameter, we were able to detect several other significantly altered parameters in regard to pupil dynamics with the use of the state of the art CIP. However, none of the changes found correlated with AMS despite the use of a well-established exposure protocol for assessment of physiological changes at high altitude in regard to AMS.

## Supporting Information

Figure S1 **Single pupillograms of one participant.** The shown pupillograms are the single puillograms of one participant on each day (A–F). In every session, measurements were repeated five times, artifact measurements were deleted and afterwards an average value was automatically calculated from the pupillograms. The average pupillogram are not shown.
(TIF)

Table S1 **Investigated parameters of the pupillary light reaction.**
(DOCX)
