# Supplementary figures and images for: Pupillary Light Reaction during High Altitude Exposure
Source: PLoS One. 2014 Feb 4;9(2):e87889. doi: 10.1371/journal.pone.0087889 (PMC3913681; doi:10.1371/journal.pone.0087889)

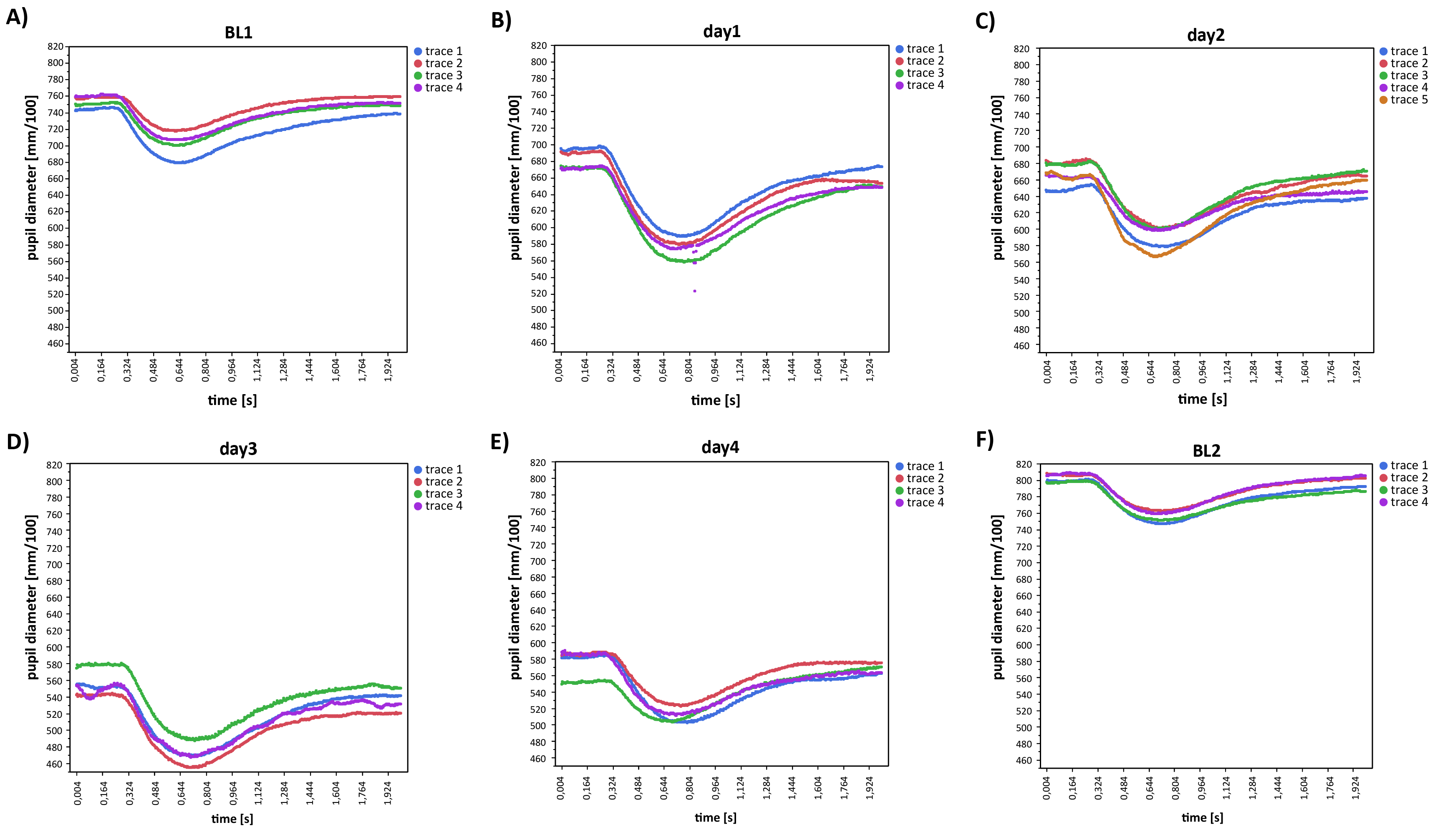

Supplement: Figure S1 — Single pupillograms of one participant. The shown pupillograms are the single puillograms of one participant on each day (A–F). In every session, measurements were repeated five times, artifact measurements were deleted and afterwards an average value was automatically calculated from the pupillograms. The average pupillogram are not shown. (TIF) [file pone.0087889.s001.tif]
