# Supplementary material for: Pupillary Light Reaction during High Altitude Exposure
Source: PLoS One. 2014 Feb 4;9(2):e87889. doi: 10.1371/journal.pone.0087889 (PMC3913681; doi:10.1371/journal.pone.0087889)
Supplement: Table S1 — Investigated parameters of the pupillary light reaction. (DOCX) [file pone.0087889.s002.docx]

**Table S1**

| ***Pupillometric parameters*** | ***Description*** |
| --- | --- |
| Initial diameter | Pupil diameter prior to stimulus |
| Amplitude | Initial diameter – minimum diameter |
| Relative amplitude | Amplitude in relation to the initial diameter |
| Latency | Time from the stimulus onset till the beginning of pupil reaction |
| Constriction velocity | Maximum velocity of pupil constriction |
